# Mental health in low-income countries: A call to improve mental health in Uganda

**DOI:** 10.1371/journal.pmen.0000313

**Published:** 2025-04-30

**Authors:** Kizito Omona, Nice Barungi Mponye Bashabire, Ritah Bulamu, Bernardine Mugabe, Samuel Ssanyu Balamaga, Betty Enyipu Akurut

**Affiliations:** 1 Faculty of Health Sciences, Uganda Martyrs University, Kampala, Uganda; 2 Counselling Department, School of Social Sciences, Uganda Christian University, Mukono, Uganda; PLOS: Public Library of Science, UNITED KINGDOM OF GREAT BRITAIN AND NORTHERN IRELAND

The World Health Organization defines mental health as a state of mental well-being that enables people to cope with the stresses of life, realize their abilities, learn well and work well, and contribute to their community [1]. It is an integral component of our general health that underpins our individual and collective abilities to make decisions, build relationships and shape the world we live in –and yet it remains a low priority in many low-income countries. Uganda, like many sub-Saharan African countries, faces significant challenges in addressing mental health concerns. The burden of mental illness is exacerbated by poverty, conflict, and a lack of awareness. Supporting and managing the wellbeing of people is faced with numerous challenges, especially for those who already face a variety of hurdles or difficulties. For example, Mugisha, Bantu & Nakalema [2] explored the mental health impact on parents raising children with Autism Spectrum Disorder (ASD) in Uganda. They highlighted stigma, economic hardship, and lack of mental health support services as key factors exacerbating mental health struggles. The results revealed very high levels of stress and stigma among parents and experiences of financial difficulties, marital conflicts, and psychological strain resulting from societal discrimination were common. Parents reported experiencing feelings of shame, embarrassment, and hopelessness, exacerbated by societal misconceptions and the lack of support systems. The prevalence of anxiety symptoms among parents (45.9%) surpassed previous estimates, indicating a significant mental health burden within this population. Another study examined the intersection of mental health and HIV/AIDS in Uganda and emphasized again, how financial stress, social stigma, and lack of resources all hinder mental well-being within this community [3]. This echoes findings in many other similar studies [4,5].

In this Opinion, we aim to provide an overview of the state of mental health in Uganda, assess existing policies and challenges, and offer possible solutions.

## 1. Prevalence and burden of mental health conditions

Studies indicate that mental health conditions are highly prevalent in Uganda, with depression, anxiety, schizophrenia, and post-traumatic stress disorder (PTSD) being among the most common conditions [6,7]. The high prevalence of PTSD in particular, is linked to the country’s history of civil conflict and displacement [8]. However, due to underreporting and lack of diagnostic tools, the true burden of mental illness is likely underestimated. An analysis from a ‘Global Burden of Disease’ study ranks Uganda among countries with the highest depression incidence rates, calling for urgent mental health reforms [9]. According to Kaagari [10], in Uganda, mental, neurological and substance use disorders are a major public health burden [10,11]. Depression, anxiety disorders, and elevated stress levels are the most common, with suicide attempts often occurring as a result [9]. Uganda is ranked among the top six countries in Africa in terms of rates of depressive disorders at 4.6% [12] while 2.9% live with anxiety disorders [13]. However this is likely to be an underestimation. In general, about 5.1% of females and 3.6% of males are affected [9].

## 2. Uganda’s mental health infrastructure and services

Uganda has limited mental health infrastructure, with the majority of psychiatric services concentrated in urban areas. The country’s primary psychiatric facility, Butabika National Referral Hospital, struggles with overcrowding and resource constraints [6]. Few general hospitals offer mental health services, and community-based psychiatric care is underdeveloped. Additionally, the ratio of mental health professionals to the population is critically low, making access to care challenging [10,14,15].

Several barriers hinder mental health service delivery in Uganda:

a) **Stigma and Cultural Beliefs**: Mental illness is often misunderstood and associated with witchcraft or spiritual possession, leading to discrimination and reluctance to seek medical treatment [16,17].
b) **Financial Constraints**: Many individuals cannot afford mental health care due to widespread poverty and the lack of insurance coverage for psychiatric services [18].
c) **Shortage of Trained Professionals**: Uganda has a severe shortage of psychiatrists, psychologists, and psychiatric nurses, limiting the availability of professional mental health care [19].
d) **Weak Policy Implementation**: Although Uganda has mental health policies, enforcement and implementation remain inadequate due to lack of political commitment and funding [10,16,17,19]. The Covid- 19 pandemic exacerbated this situation [20].

## 3. Current interventions and policy framework

Despite grappling with numerous challenges [17–19], Uganda has made some progress in addressing mental health through various initiatives:

a) The integration of mental health services into primary healthcare facilities aims to improve accessibility [21].
b) Non-governmental organizations (NGOs) and community-based programs have played a crucial role in raising awareness and providing psychological support [6,10,16–18,21].
c) The Mental Health Act of 2019 seeks to improve the legal framework for mental health care delivery, though implementation remains slow [4,21].

## 4. Recommendations for improvement

To enhance mental health care in Uganda, we call for the following measures to be considered as a priority for funders and policy makers:

1) **Increased Funding**: Allocating more resources to mental health research, programs and infrastructure.
2) **Training and Capacity Building**: Expanding training programs for mental health professionals and integrating mental health education into medical and nursing curricula.
3) **Community-Based Approaches**: Strengthening local mental health support systems, including peer counseling and traditional healer collaboration with healthcare providers.
4) **Policy Strengthening and Implementation**: Ensuring that mental health policies are effectively enforced and adequately funded.
5) **Public Awareness Campaigns**: Addressing stigma through education and advocacy to encourage help-seeking behavior.

Addressing all these challenges requires comprehensive support systems, including training programs, workshops, and access to resources aimed at empowering people and enhancing their well-being. Interventions can be developed to alleviate the burdens faced, ultimately improving quality of life. We must work to fully understand and address the specific stressors and effective coping mechanisms within the communities.

## 5. Conclusion

Mental health in Uganda remains a significant public health challenge, with numerous barriers limiting access to care. However, with strategic policy changes, increased funding, and community-driven initiatives, the country can improve mental health outcomes. Strengthening Uganda’s mental health system will not only improve individual well-being but also contribute to broader social and economic development.
